# Complete Genome Sequence of the Macrolide-Resistant *Mycoplasma pneumoniae* Strain C267 in China

**DOI:** 10.1128/genomeA.00236-16

**Published:** 2016-04-07

**Authors:** Shaoli Li, Hongmei Sun, Fei Liu, Hanqing Zhao, Baoli Zhu, Na Lv

**Affiliations:** a Capital Institute of Pediatrics, Beijing, China; b Institute of Microbiology, Chinese Academy of Sciences, Beijing, China

## Abstract

Macrolide-resistant *Mycoplasma pneumoniae* strains can cause severe *M. pneumoniae* pneumonia in children. Here, we report the complete genome sequence of the macrolide-resistant *M. pneumoniae* strain C267, deposited in GenBank under accession number CP014267, which provides new insights for other potential macrolide-resistant mechanisms in *M. pneumoniae* clinical isolates in China.

## GENOME ANNOUNCEMENT

Macrolide-resistant *Mycoplasma pneumoniae* (MRMP) strains, which are defined by point mutations in 23S rRNA genes, have increased rapidly worldwide since 2000 (1, 2), especially in South Korea, China, and Japan (3–5). MRMP can cause severe *M. pneumoniae* pneumonia in children (1). But why have *M. pneumoniae* resistance rates been so high in Asia? To answer this question, there is a need for whole-genome sequencing of MRMP isolated from Asia to fully comprehend its mechanism.

The macrolide-resistant *M. pneumoniae* strain C267 was isolated in China and displays high MIC values (>512 µg/ml) to erythromycin and azithromycin (testing was carried out according to CLSI document M43A). To explore the genomic variations that are potentially involved in its macrolide resistance, we used the Illumina HiSeq 2000 platform for whole-genome sequencing. A total of 1,124,693 paired-end reads, with an average read length of 116 bp, were generated. Raw reads were first filtered using the DynamicTrim and LengthSort Perl scripts provided in the SolexaQA suite and then assembled using SOAPdenovo (http://soap.genomics.org.cn), yielding 16 scaffolds with an average length of 50,331 bp. Gaps were closed by PCR and subsequently analyzed with an ABI-3730 genetic analyzer (Applied Biosystems, USA). NCBI’s Prokaryotic Genome Annotation Pipeline annotated the complete genome. It comprises 816,498 bp of chromosomal DNA (40.0% GC content), containing 714 coding sequences, 1 rRNA operon, and 37 tRNAs. SOAPsnp was used to score single-nucleotide polymorphisms (SNPs) from aligned reads (6). In total, 352 SNPs were identified with reference genome M129.

SNPs associated with macrolide resistance were first identified to cluster in the macrolide-specific efflux system gene *macB*, which encodes a macrolide ATP-binding cassette (ABC) transporter protein according to a BLAST analysis against information in the Antibiotic Resistance Database (ARDB). This implies that the mutants of the efflux system might be involved in MRMP.

### Nucleotide sequence accession number.

The complete genome sequence of *M. pneumoniae* strain C267 has been deposited in GenBank under accession number CP014267.
